# Synthesis and Evaluation of Biological Activity of Antimicrobial – Pro-Proliferative Peptide Conjugates

**DOI:** 10.1371/journal.pone.0140377

**Published:** 2015-10-16

**Authors:** Paulina Kosikowska, Michal Pikula, Paulina Langa, Piotr Trzonkowski, Michał Obuchowski, Adam Lesner

**Affiliations:** 1 Department of Biochemistry, Faculty of Chemistry, University of Gdansk, Gdansk, Poland; 2 Department of Clinical Immunology and Transplantology, Medical University of Gdansk, Gdansk, Poland; 3 Department of Medical Biotechnology, Intercollegiate Faculty of Biotechnology UG-MUG, Medical University of Gdansk, Gdansk, Poland; University of Kiel, GERMANY

## Abstract

Skin represents the largest organ of the human body and plays a crucial role in its protection from the negative impact of the outside environment, maintains its homeostasis, enables sensory interaction and thermoregulation. The traumatized skin tissue undergoes several phenotype switches due to progressive reoxygenation and release of cytokine and growth factors, that activate mechanisms of reparative processes. However, in case of wounds colonized with pathogenic microflora natural regenerative mechanisms become substantially impaired, that could lead to chronic inflammatory states with non-healing skin lesions. Herein, we present the initial results of our studies aimed at the design of bifunctional peptide-based compounds. The chemical approach, that was utilized in this work, was based on the conjugation of antimicrobial peptides with the peptides, that have potential pro-proliferative and/or cytoprotective activity towards human keratinocytes and fibroblasts, in order to obtain antimicrobials with reduced cytotoxicity or compounds that maintain both activities, i.e. inhibit bacterial or fungi growth and activate cell proliferation/migration in *in vitro* tests. As a result, we obtained a group of peptide conjugates that effectively inhibited the growth of selected bacterial and fungi strains and were able to stimulate proliferation and migration of keratinocytes and fibroblasts under their effective microbicidal concentrations.

## Introduction

Progressive spread of bacterial and fungi infections is one of the main problems in treatment of chronic wounds. The simplified definition of chronic wound refers to ulcer older than three month of age with disturbed mechanism of healing which disables rebuilding of injured tissue [1] According to the etiology and most typical localization chronic wounds could be divided into three main groups: venous leg ulcers, diabetic foot ulcers, and pressure ulcer. Additional factors predisposing to the impaired healing arise from prolonged inflammatory states, ischemia, immunosuppressive therapies, cancer or advanced age [2].

Skin wound healing is a very complex and highly regulated biochemical process that starts immediately after the initial lesion and proceeds through three main phases overlapping in time: inflammation, proliferation and remodeling. Each phase is well orchestrated by specific interactions between various cytokines, growth factors and proteases [3]. Nevertheless, the whole process could take weeks or months depending on the type of injury and individual factors affecting the wound healing.

Due to the persistent presence of different type of microorganisms as the components of the human skin microflora it is almost impossible to keep the wound area completely sterile. Thus, wound microbiology could be characterized by three interconnected stages: contamination, colonization and infection. The first step relates to the presence of small amount of non-replicating microorganisms without any evidence of the induced wound damage. This step could further proceed to colonization, when bacteria start growing and replicating increasing their load within the wound.When colonization reaches the critical level, so that the host immune response cannot fully control this, the healing process becomes affected. The transition to infection stage occurs when microorganisms start invading dermal or sub-dermal area and clinical manifestations of inflammation like pain, heat, erythema, pruritus and purulent exudates are observed [4]. Thus, the balance between host response and bacteria proliferation within the wound decides about the shift from the step of contamination to colonization or fully developed infection of wounded skin. That is why impaired self-defense activity of injured skin often leads to chronic non-healing inflammatory states.

Despite the significant achievements in medical care of wounds, polymicrobial nature of non-healable ulcers make them especially difficult to cure. Due to the low oxygen level in most of chronic wounds, they contain not only aerobic strains, like *Staphylococcus aureus*, *Enterococcus faecalis*, *Enterobacter cloacae*, *Pseudomonas aeruginosa*, *Proteus* spp., but also anaerobes, like *Bacteroides*, *Prevotella*, *Porphyromonas* spp., which presence is usually underestimated due to more complicated diagnostics [5]. Additional problem arises from the increasing resistance of pathogenic bacteria and fungi to conventional antibiotic therapies, combined with lack of novel antimicrobial agents with alternative mechanism of action [6].

In the last century pharmaceutical industry was mostly focused on the discovery of small compounds isolated from the natural sources or obtained by means of rational molecular design and extensive screening of chemical libraries. For a long time bioactive peptides and proteins were excluded from the group of potent drug candidates due to their low oral bioavailability and relatively high molecular weight (above 500 Da). However, the intensive development of genomic and proteomic technologies have cardinally changed the previous approach in drug discovery showing the potency of biopharmaceuticals as effective and highly specific therapeutics of asthma, cancer, neuropathic pain, HIV, heart disease, stroke and diabetes [7].

Like in case of other biologically active compounds, nature offers the most abundant source of leads for the discovery of peptide-based drugs. Being a product of the evolutionary selection, endogeneous peptides utilize diverse mechanism responsible for their biological activity, including electrostatic attraction to negatively-charged membranes of microorganisms or certain cancer cells [8,9], specific receptor-mediated targeting, interfering with intracellular biochemical reactions (DNA/RNA synthesis, enzyme inhibition) [10,11]. Such multitarget affinity of AMPs *in vivo* determines not only its antimicrobial activity, but is also responsible for analgetic, wound healing, antioxidant, immunomodulatory or antihypertensive properties of the compounds [12,13]. Moreover the results of long-term researches revealed that more than one type of *in vivo* activity could be attributed to many endogenous peptides [10]. However, it should be also mentioned that due to multifaceted functionality of bioactive peptides their therapeutic application is often hampered or limited because of the risk of undesirable interactions.

In our work we decided to focus on antimicrobial and pro-proliferative activities of the peptides, as we consider them as the most crucial for the stimulation and support of natural skin regenerative processes. The main idea of our preliminary studies was to test the possibility of obtaining relatively short (up to 20 residues) bifunctional peptide derivatives of simple amino acid composition by chemical linkage of two peptides with the defined antimicrobial and wound healing activity. To do so, we selected several compounds from the available literature data (Table A in S1 File) with experimentally confirmed biological activity and bind them together by means of small biologically inert and non-immunogenic PEG-linker (8-amino-3,6-dioxaoctanoic acid).

Selection of antimicrobial peptides was done applying simple criteria of short amino acid composition (up to 12 amino acids), linear structure (lack of disulfide bridges), positive net charge, established antibiotic properties towards wide spectrum of bacteria and low hemolytic activity. Therefore, two Lys-rich derivatives of amphibian AMP temporin-1CEb and one synthetic peptide KSLW were chosen.

The fragments of conjugates that were supposed to define their pro-proliferatory or/and cytoprotective properties were selected among the compounds with short amino acid sequences and confirmed wound-healing potential. Thus, endogenous dipeptide carnosine (CAR), synthetic Leu-enkephalin analogue dalargin (DAL) and hexapeptide (CTEN2), derived from the sequence of extracellular matrix glycoprotein tenascin X, were proposed.

The obtained peptide conjugates were further tested *in vitro* towards human primary dermal fibroblasts and human keratinocyte cell line in order to evaluate their pro-proliferative and pro-migratory activity, and towards several bacteria and fungi strains to examine their antimicrobial properties.

## Materials and Methods

### Peptide synthesis

All mentioned peptides were synthesized manually by means of the solid phase method applying Fmoc (fluorenyl-9methoxycaronyl) chemistry under the standard conditions. 2-chlorotritylchloride (substitution 0.48 meq/g, GL Biochem, Shanghai Ltd.) and S RAM (substitution 0.23 meq/g, RAPP Polymere, Germany) resins were used as a solid support. Fmoc-protected amino acids were attached to the resin using N,N’-diisopropylcarbodiimide (DIPCI) and N-hydroxybenzotriazole (HOBt). After deprotection with 20% piperidine in a mixture of DMF/NMP (1:1, v/v), the peptide chain was elongated applying the same DIC/HOBt method using a three-fold molar excess of Fmoc-protected amino acids or PEG-fragment (Fmoc-O2Oc-OH, Iris Biotech, Germany) over the resin active sites. Cleavage of the peptides from the resin was done using TFA/phenol/triisopropylsilane/H_2_O mixture (88:5:2:5,v/v). Purity of the peptides was further analyzed by reverse phase high performance chromatography (RP-HPLC) on Varian Prostar HPLC System equipped with Aeris Widepore 3.6 μm XB-C18 (100 x 4.60 mm) column (Phenomenex, USA) and UV-VIS detector. A linear gradient from 10 to 90% B within 40 min (A: 0.1% TFA; B: 80% acetonitrile in A) with a flow rate 1 mL/min was employed. The peptides were monitored at 226 nm. Mass spectra of the synthesized peptides were recorded using a Biflex III MALDI TOF mass spectrometer (Bruker, Germany) and α-cyano-4-hydroxycinnamic acid (CCA and/or 2,5-dihydroxybenzoic acid (DHB) used as matrix.

### Circular dichroism

Circullar dichroism spectra of analyzed compounds were recorded on a Jasco J-815 spectropolarimeter using a quartz cell of 1 mm path length between 195–260 nm at room temperature. Peptides at concentration of 0.4 mg/ml were dissolved in water or 30 mM SDS solution. SDS micelle system was used as the simplified model of negatively charged lipid membrane [14]. On the basis of the obtained results the alpha-helical content was determined from the mean residue ellipticities at 222 nm according to Eq 1:

$$\%\;\text{Helix}\;=\;({\left[\theta \right]}_{\text{obs}}/\left({\left[\theta \right]}_{helix}\;\text{x}\;(1-2.57/l)\right)\;\text{x}\;100,$$ (1)

where [θ] _obs_ is the experimentally observed mean-residue ellipticity at 222 nm, [θ]_*helix*_ is the theoretical ellipticity of peptide of infinite length with a 100% helix population, taken as -39 500 deg·cm^2^/dmol, and *l* is the number of peptide bonds [15].

### Antimicrobial activity

*Escherichia coli* (PCM 2057), *Staphylococcus aureus* (PCM 2054), *Staphylococcus epidermidis* (PCM 2118), *Pseudomonas aeruginosa* (PCM 499) were obtained from Polish Collection of Microorganisms. Fungi strains *Candida albicans* (CCM 8186), *Candida glabrata* (CCM 8270), *Candida parapsilosis* (CCM 8260), *Candida krusei* (CCM 8271), *Candida tropicalis* (CCM 8264) were acquired from Czech Collection of Microorganisms.

Luria-Bertani broth (LB) and Sabouraud Dextrose broth (SB) (Biocorp, Poland) were used for cultivation of bacteria and fungi strains, respectively. Antimicrobial activity of peptides was analyzed through determination of MIC (minimal inhibitory concentration) parameter according to the standard microdilution technique performed on 96-well plates. Mueller-Hinton broth (MHB) was used as the working medium for all bacterial strains, while in case of fungi it was additionally supplemented with 2% glucose.

Briefly, inoculum was prepared from freshly grown cultures of microorganisms being at their exponential phase of growth. Each well of 96-well plates containing 100 μL of serially diluted peptides in PBS (phosphate buffer saline) at concentration ranging from 2 to 250 μg/mL were inoculated with 100 μL of approximately 10^5^ CFU/mL of bacterial suspension or about 2.5 x 10^3^ CFU/mL of fungi suspended in a double concentrated Mueller-Hinton broth (supplemented with 2% glucose for *Candida* spps.). Then plates were incubated overnight at 37°C and absorbance was read at 590 nm after 24 h. Cultures without peptide were treated as positive control, while uninoculated Mueller-Hinton broth was defined as negative control. All measurements were run at least in triplicate. MIC is defined as the minimal concentration that completely inhibits visible growth of microorganisms.

### Isolation of human dermal fibroblasts

Biopsies of healthy human skin were obtained aseptically from three donors (47–69 years old) undergoing circumcision after obtaining their written informed consent. All the procedures were approved by the Ethics Committee of Medical University of Gdansk (NKEBN/483/2011. Issued: 2011-12-19). The samples were washed several times with sterile PBS (Sigma-Aldrich, Germany) containing antibiotic (200 U/mL penicillin and 200 μg/mL streptomycin), after which, the subcutaneous tissue was cut off with a sharp blade. The resulting sheets were cut into small fragments, washed two times with sterile PBS and treated with 6 U/mL dispase II (BD, USA) overnight at 4°C to separate epidermis and dermis. The dermis was then digested with 1.0 mg/mL collagenase type I (from Clostridium histolyticum Sigma-Aldrich, Germany) for 2 hours at 37°C to isolate fibroblasts. Cells were used between the 2nd and 4th passage in all experiments.

### Cell culture conditions

In our study two kind of cells were used: immortalized human HaCaT keratinocytes (DKFZ Heidelberg, Germany) and primary dermal fibroblasts obtained from 3 healthy donors. Both types of the used cell lines were grown in Dulbecco's modified Eagle's medium (DMEM) (Sigma–Aldrich, Germany), that contained 4500 mg/mL of glucose, 584 mg/mL of L-glutamine, sodium pyruvate and sodium bicarbonate. Additionally, medium was supplemented with 10% of FCS (fetal calf serum, Sigma-Aldrich, Germany), 100 U/mL of penicillin and 100 μg/mL streptomycin (Sigma-Aldrich, Germany). The cells were grown in culture dishes (BD, USA) under standard conditions in a humidified atmosphere with 5% CO_2_ at 37°C and medium was changed every 2 days.

### Cell proliferation assay

The fibroblasts and keratinocytes were seeded at density of 4500 and 6000 cells per well, respectively, into 96-well plates suspended in a medium supplemented with 10% of FCS. After 24 h the media were exchanged with serum- and antibiotic- free DMEM and tested peptides dissolved in PBS were added yielding final concentration ranging from 1 to 50 μg/mL. Cells were incubated for the next 24 hours, when the medium was exchanged again and fresh portion of peptides (used at the same concentrations as previously) was added. Incubation was continued for the next 48 hours (the overall time of cell exposition to peptide was 72 h). At the end of the experiment cells were treated with 20 μL of MTT (5 mg/mL in PBS). After 3 h at 37°C under 5% CO_2_ medium was discarded and replaced by 6mM HCl in isopropanol. After 15 min when the purple crystals of formazan were fully solubilized, absorbance was read at 570 nm with a plate reader. The optical density reflects the level of cell viability as MTT is converted in purple formazan product only in the presence of viable mitochondria of the living cells.

### Cell migration assay

Fibroblasts and keratinocytes were seeded (5x10^5^ cells/well) in 6-well plates. When desired monolayer confluence was reached, cells were starved for 24 hours in serum-free DMEM. The monolayer was scratched then with 200μL-pipette tip. The floating cells and cell debris were removed by double washing in PBS, further replaced with a fresh serum-free medium. Wounded monolayers were incubated for the next 24 h in the presence of tested peptides used at concentration of 25 μg/mL and 5 μg/mL of Mitomycin C (Sigma Aldrich) to prevent proliferation. At the end of the experiment medium was removed and the cells were fixed with 3.7% PFA for 5 min, stained for the next 30 min with 0.05% crystal violet solution, washed several times with distilled water and the plates were left inverted for a couple of minutes to drain. The wounded areas were then photographed by means of Zeiss Observer D1 microscope equipped with camera AxioCam and analyzed with AxioVision software (Carl Zeiss, Germany). Migration activity of the cells treated with the tested peptides were expressed as the percentage of the wounded area with respect to the control probe (cell cultures without any of the peptide), assumed as 100%.

### Statistics

Experimental data were processed by means of GraphPad Prism 5 software. Statistical analysis were performed according to the one-way ANOVA test for repeated measurements and *post hoc* Tukey test with significance level alpha set as 0.05 (P < 0.05)

## Results

### CD spectra of the peptides

Circular dichroism method was applied to obtain a basic information about the secondary structure of the synthesized compounds (with the exception of CAR, CAR3, DAL, CTEN2) in two chemical environments, water and 30 mM SDS. As expected, all the tested compounds in water adopted a random-coil structure, while in the presence of 30 mM SDS most of them formed α-helices with characteristic negative minima around 208 and 220 nm. Distortion of the helical structure in 30 mM SDS was observed for KSLW peptide and its conjugate DAL-PEG-KSLW. The examples of CD spectra for selected peptides could be seen in supporting information (Figure A in S1 File). On the basis of the experimentally obtained values of the mean residue ellipticities of peptides at 222 nm their alpha-helical content was calculated according to the Eq 1 (Table 1).

**Table 1:** Primary structures of the peptides with their basic physicochemical characteristics.

No	Compound	Primary sequence	MW (calc./obs.)	Net charge	α-helicity^a)^[%]	Mean hydrophobicity^b)^	RT [min]
1	DK5	IKKILS***K***IKKLL-NH_2_	1424.2/1423.9	+6	29	0.383	10.98
2	LK6	IKKILSKIKKLLK-NH_2_	1552.1/1552.5	+7	27	0.054	10.52
3	KSLW	KKVVFWVKFK-NH_2_	1307.7/1307.7	+5	12	0.170	10.06
4	DAL	Y***A***GFLR	725.8/726.1	+2	ND^c^	0.367	10.20
5	CAR	βAH	226.1/227.6	+0.1	ND	-0.7	3.67
6	CAR3	βAHβAHβAH	641.3/643.1	+0.3	ND	-0.7	5.72
7	CTEN2	EGLEPG	600.6/600.9	-2	ND	-0.933	2.87
8	DK5-PEG-DAL	IKKILS***K***IKKLL-**PEG**-Y***A***GFLR	2277.9/2279.2	+7	20	0.378	12.39
9	DAL-PEG-DK5	Y***A***GFLR-**PEG-**IKKILS***K***IKKLL-NH_2_	2276.9/2278.2	+7	35	0.378	16.94
10	CAR-PEG-DK5	βAH-**PEG-**IKKILS***K***IKKLL-NH_2_	1777.3/1778.7	+6.1	41	0.229	14.14
11	CAR3-PEG-DK5	ΒAHβAHβAH-**PEG-**IKKILS***K***IKKLL-NH_2_	2192.4/2194.8	+6.1	27	0.022	13.35
12	DAL-PEG-LK6	Y***A***GFLR-**PEG-**IKKILSKIKKLLK-NH_2_	2405.1/2405.9	+8	25	0.153	17.27
13	CTEN2-PEG-LK6	EGLEPG**-PEG-**IKKILSKIKKLLK-NH_2_	2279.9/2281.3	+5	20	-0.258	15.85
14	CTEN2-PEG-KSLW	EGLEPG**-PEG-**KKVVFWVKFK-NH_2_	2035.4/2036.6	+3	27	-0.244	10.38
15	DAL-PEG-KSLW	Y***A***GFLR-**PEG-**KKVVFWVKFK-NH_2_	2161.6/2161.8	+6	22	0.244	13.04

^a)^ Alpha-helical content was calculated according to the Eq 1 on the basis of the results obtained for peptides dissolved in 30 mM SDS

^b)^ Mean hydrophobicity values were calculated by means of ProtParam program [16] as the sum of hydropathy values of all the amino acids, divided by the number of residues in the sequence (PEG fragment was excluded from the calculations)

^c)^ ND—not determined

D-analogues of the amino acids are marked as italic type

### Analysis of antimicrobial properties

All peptides were screened against the wide range of microorganisms, including Gram-negative, Gram-positive bacteria and fungi from *Candida* species (Table 2). Broth microdilution method for MIC determination was used as a standard procedure. All assays were performed on liquid Mueller-Hinton broth, which was additionally supplemented with 2% glucose when fungicidal activity towards *Candida* spps. was analyzed.

**Table 2:** Antimicrobial activity of the peptides.

.		MIC [μg/mL]											
No	Compound	*C*. *albicans*	*C*.*krusei*	*C*. *parapsilosis*	*C*.*tropicalis*	*C*.*glabrata*	*B*. *subtilis*	*B*. *cereus*	*S*. *epidermidis*	*S*.*aureus*	*E*.*coli*	*P*.*mirabilis*	*P*. *aeruginosa*
1	**DK5**	31.3	7.8	31.3	< 2	62.5	15.6	>125	7.8	>125	>125	NI ^a)^	50
2	**LK6**	31.3	7.8	7.8	<2	31.3	7.8	50	7.8	100	>125	NI	25
3	**KSLW**	7.8	7.8	2.0	<2	15.6	7.8	50	7.8	25	7.8	NI	25
4	**DAL**	NI											
5	**CAR**	NI											
6	**CAR3**	NI											
7	**CTEN2**	NI											
8	**DK5-PEG-DAL**	100	7.8	2.0	7.8	>125	7.8	>125	7.8	>125	7.8	NI	>125
9	**DAL-PEG-DK5**	25	3.9	2.0	<2	125	2.0	25	7.8	50	25	NI	25
10	**CAR-PEG-DK5**	15.6	3.9	31.3	<2	62.5	3.9	100	3.9	125	100	NI	50
11	**CAR3-PEG-DK5**	100	7.8	2.0	7.8	125	15.6	>125	7.8	>125	>125	NI	50
12	**DAL-PEG-LK6**	25	7.8	2.0	3.9	>125	7.8	12.5	7.8	25	25	NI	25
13	**CTEN2-PEG-LK6**	125	15.6	125	7.8	>125	7.8	>125	7.8	>125	>125	NI	>125
14	**CTEN2-PEG-KSLW**	>125	>125	>125	15.6	>125	>125	>125	7.8	>125	>125	NI	>125
15	**DAL-PEG-KSLW**	31.3	31.3	62.5	7.8	125	15.6	125	3.9	62.5	7.8	NI	62.5

^a)^NI—no inhibitory activity

According to the obtained results, none of the native wound-healing peptides (DAL,CAR, CAR3, CTEN2) was able to inhibit bacterial or fungi growth.

The most sensitive Gram-positive strain was *S*. *epidermidis* with established MIC below 10 μg/mL both for the tested native AMPs as well as their conjugates. Similar sensitivity was observed for *B*. *subtilis*, which growth was inhibited by almost all compounds used in the range of 2–15.6 μg/mL, with the exception of conjugate CTEN2-PEG-KSLW, being inactive towards selected microorganisms. The most pathogenic Gram-positive bacteria from this group, i.e. *S*. *aureus*, was rather moderately sensitive, with the MICs of 25 μg/mL established for KSLW and DAL-PEG-LK6 compounds, and 50 μg/mL for DAL-PEG-DK5.

In case of Gram-negative strains, *P*. *mirabilis* was found completely resistant to all tested compounds. The growth of *E*.*coli* and *P*.*aeruginosa* was inhibited by KSLW (MIC of 10 and 25 μg/mL, respectively), and two conjugates DAL-PEG-DK5 and DAL-PEG-LK6 (25 μg/mL in both cases).

In comparison to the mixed response of bacteria, growth ability of selected *Candida* strains was substantially hampered by the tested compounds. The most sensitive *C*. *tropicalis* strain was extremely susceptible to the effect of every analyzed AMPs and their conjugates introduced to the incubation medium (MIC below 3.9 μg/mL). Again, the most pronounced antifungal activity was observed for KSLW, DAL-PEG-DK5 and DAL-PEG-LK6.

### Analysis of pro-proliferative activity

**Fig 1:**
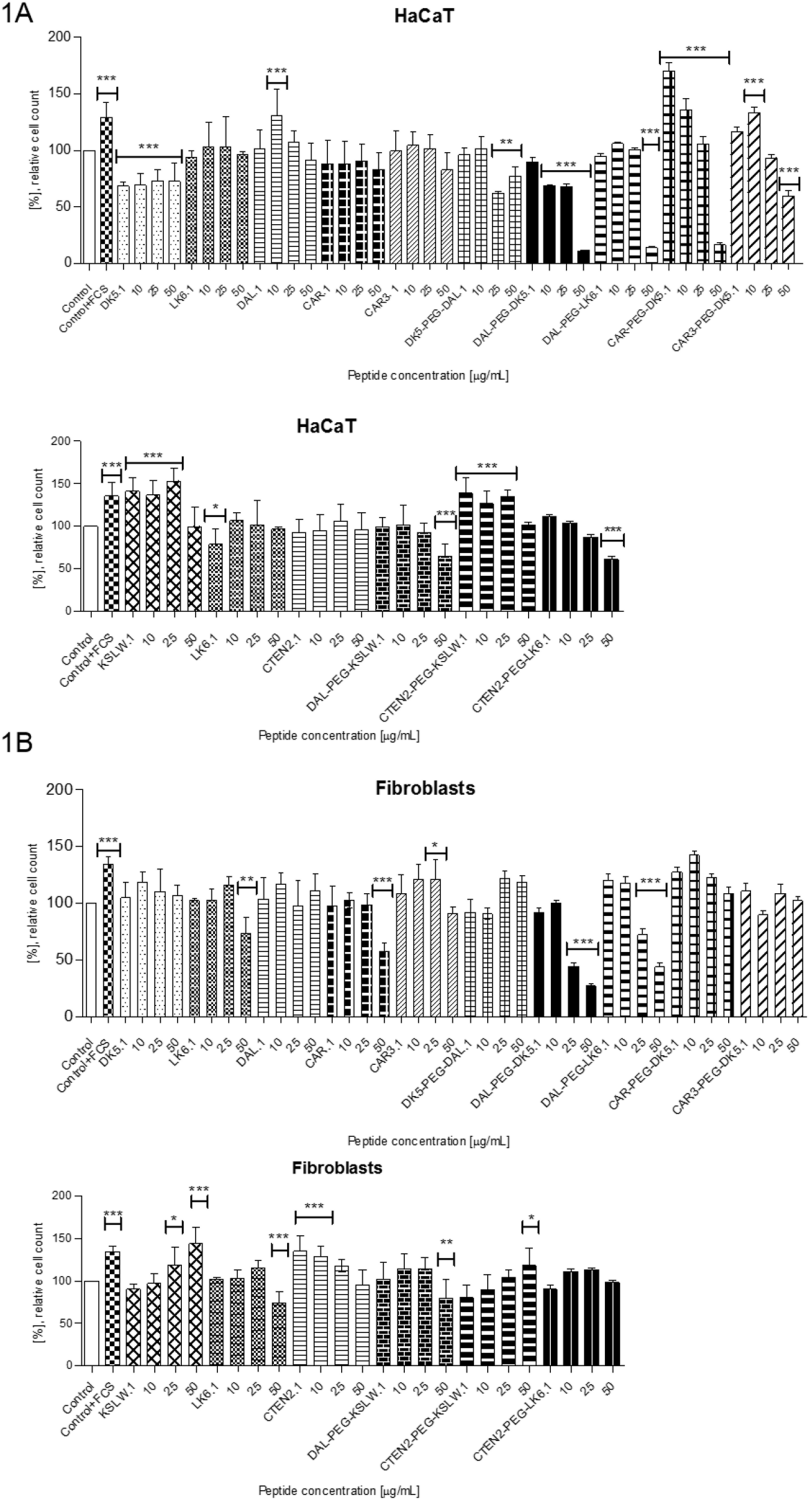
Pro-proliferative activity of the tested peptides towards human keratinocytes (A) and fibroblasts (B). Cells were incubated for 72 hours in serum-free DMEM medium in the presence of peptides applied at concentrations of 1,10, 25 and 50 μg/mL, respectively. The data represents results of MTT assay expressed as the percentage of the cell viability in comparison to their control sample (cells incubated in serum-free DMEM without tested peptides), additional positive control corresponds to the cells incubated in the presence of 10% FCS. All experiments were performed independently in triplicate. *P < 0.05, **P < 0.01, ***P < 0.001 vs. control.

Pro-proliferative properties of the tested compounds was analyzed towards immortal human keratinocyte cell line (HaCaT) and fibroblasts obtained from healthy human skin biopsies (Fig 1A and 1B). [17,18].

In order to evaluate a long-term effect of peptides, experiments were carried out for 72 hours. Before addition of the tested compounds, the cell medium was exchanged for the fresh one not containing any serum or antibiotic.

Analyzing the effect of the tested peptides on proliferation of HaCaT cell line, we observed a negative dose-response relationship in case of the native peptide DK5 and its conjugates (DAL-PEG-DK5, DK5-PEG-DAL) with the lowest cell viability for DAL-PEG-DK5 used at 50 μg/mL. The same cytotoxic effect at 50 μg/mL was detected for DAL-PEG-LK6. However, when DK5 was linked with CAR and CAR3 we noticed a substantial increase in cell count at concentrations ranging from 1 to 10 μg/mL, nevertheless, at 50 μg/mL compounds were cytotoxic.

The native KSLW peptide, as well as its conjugate CTEN2-PEG-KSLW, exerted a noticeable pro-proliferatory activity when used at concentrations of 1–25 μg/mL.

The native wound-healing peptides (DAL, CTEN2, CAR, CAR3) were found to be non-toxic in the whole range of concentrations (1–50 μg/mL), however, their pro-proliferative effect on keratinocytes was rather moderate.

In case of fibroblasts, the lowest cell viability was, again, observed for DAL-PEG-DK5 and DAL-PEG-LK6 introduced at 25 and 50 μg/mL, however, the native DK5 and LK6 peptides were non-toxic under the same test conditions.

As for the native KSLW peptide and its conjugate CTEN2-PEG-KSLW we observed a dose-response relationship, with the highest cell count when compounds were applied at 50 μg/mL. Increased cell count was also detected for CTEN2 peptide used at 1–25 μg/mL. The rest of compounds were found non-toxic towards fibroblast cell line with the absorbance of MTT test similar to the control probe.

### Analysis of pro-migratory properties

An *in vitro* scratch test was performed to investigate the effect of the analyzed compounds on keratinocytes and fibroblasts migration. Evaluation was based on the efficiency of the invasion of the monolayer cells to the prewounded area within 24 h after stimulation with the appropriate compound added at concetration of 25μg/mL.

**Fig 2:**
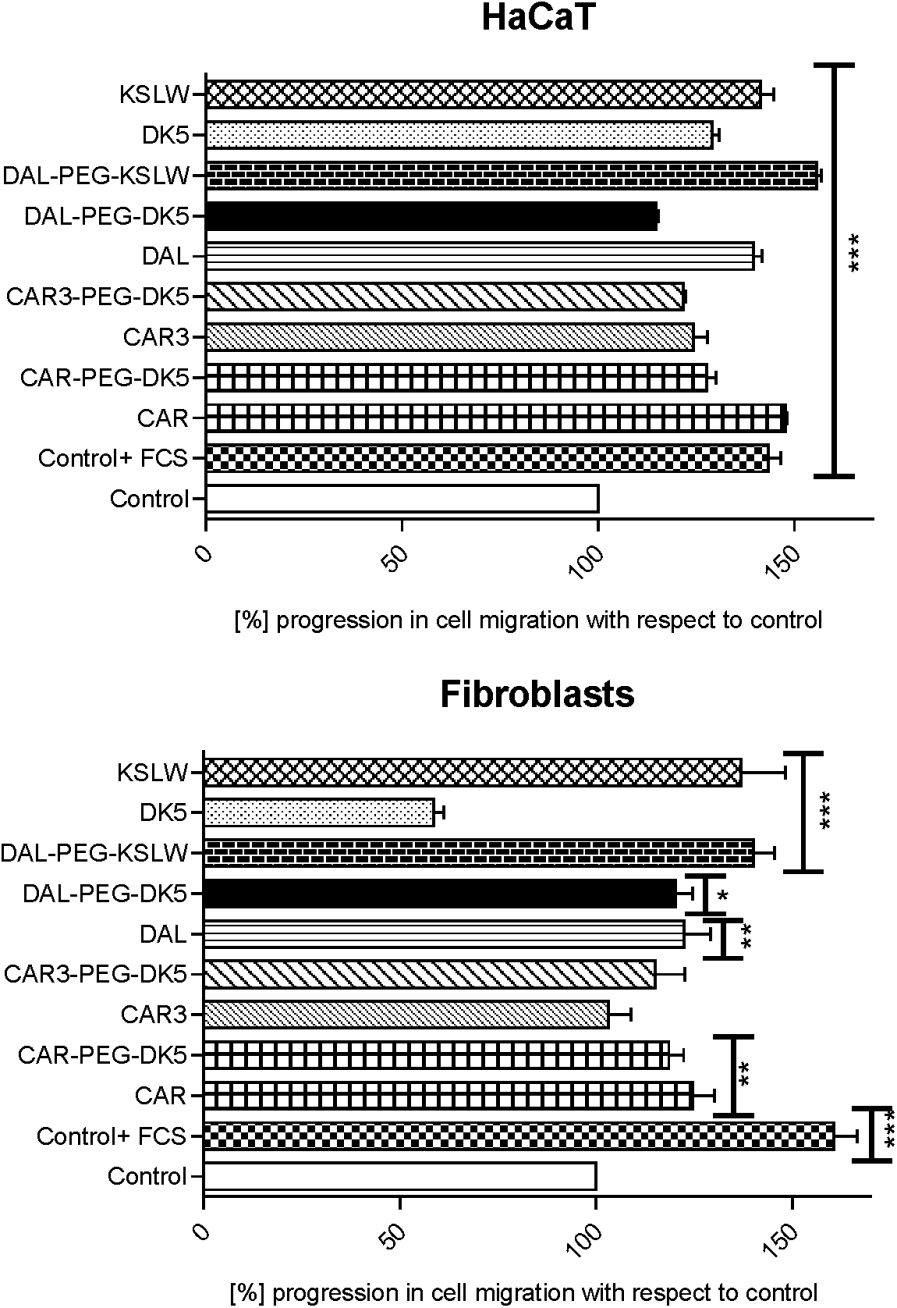
Effect of the peptides on HaCaT keratinocytes and fibroblasts cell migration in the *in vitro* scratch test. HaCaT cells and fibroblasts were grown in 6-well plates until the 100% confluence was reached, then cells were starved for the next 12 hours in DMEM without 10% FCS and scratched once vertically with a 200μL pipette tip. After being washed two times with sterile PBS, a fresh portion of DMEM medium was added and cells were treated with the tested peptides applied at their optimal doses (25 μg/mL). Migration was analyzed after 24 hours by means of Zeiss Observer D1 microscope, wounding areas were analyzed with AxioVision software and expressed as the percentage of the wound width in comparison to the control sample (cells incubated in DMEM without FCS). Control+ FCS corresponds to the sample with cells incubated in the medium containing 10% FCS and was treated as the additional positive control. Data represent the mean ± SD of three independent experiments. *P < 0.05, **P < 0.01, ***P < 0.001 vs. control.

**Fig 3:**
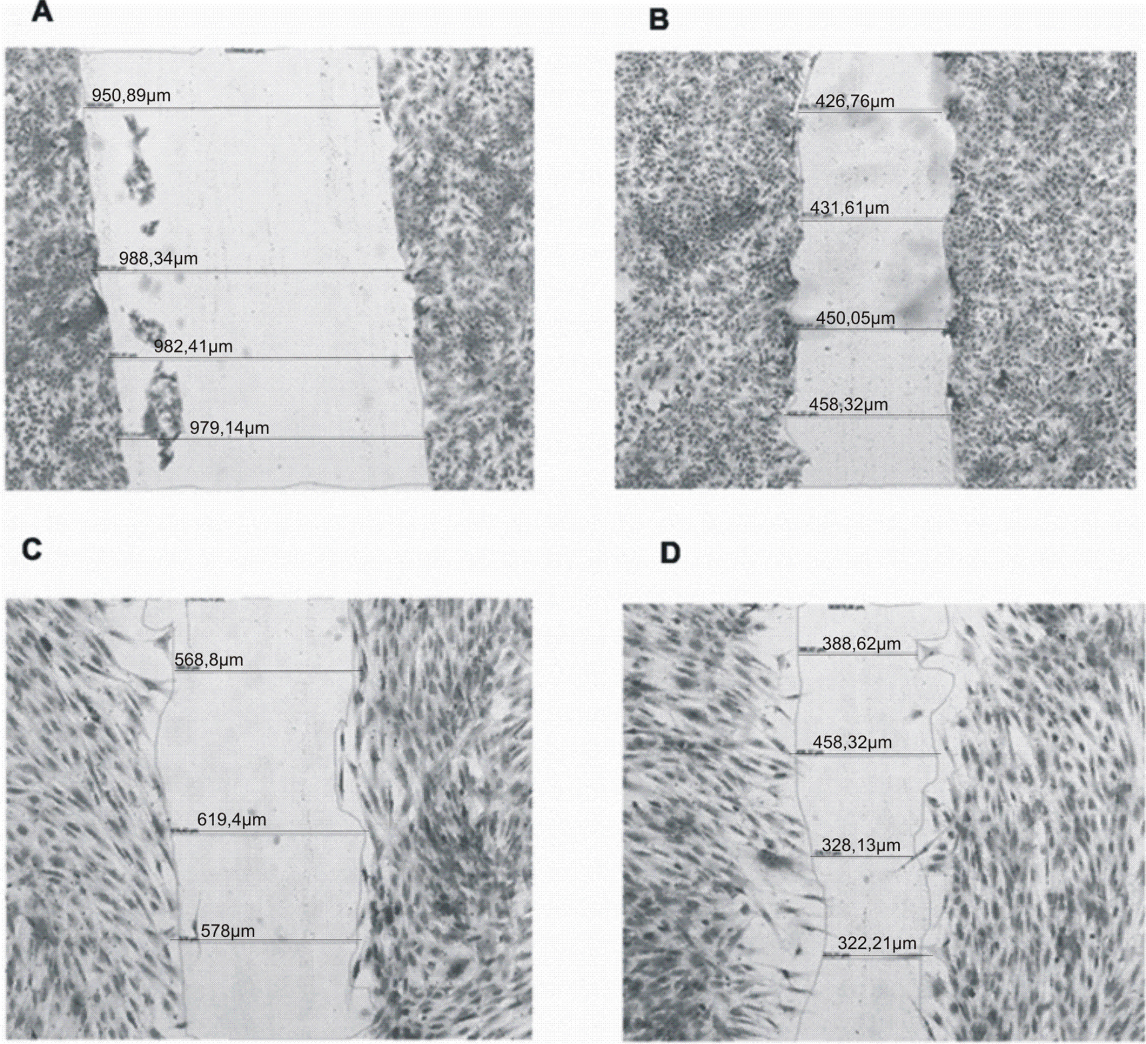
Stimulating effect of DAL-PEG-KSLW peptide on the migration of keratinocytes and fibroblasts after 24 hours of incubation. A—control probe for keratinocytes, B—migration of keratinocytes in the presence of the peptide (25 μg/mL), C—the control probe for fibroblasts, D- migration of fibroblasts in the presence of the peptide (25 μg/mL). HaCaT cells and fibroblasts were grown on the 6-well plates to confluence. Then cells were serum-starved for the next 12 hours. After that, the medium was changed and cells were stimulated with the tested peptides applied at concentration of 25 μg/mL. Incubation was continued for the next 24 hours, then cells were fixed, dyed with 0.05% of crystal violet and analyzed using Zeiss Observer D1 microscope.

As illustrated in Fig 2 all of the tested peptides had a pro-migratory effect on the keratinocytes, with the best results obtained for KSLW, DAL-PEG-KSLW and CAR. Migration of the cells in this case was comparable to the effect induced by 10% FCS and more pronounced than in case of the control probe. It is worth of mentioning, that migration stimulated by the conjugate DAL-PEG-KSLW (Fig 3) was about 15% higher than that observed for the native peptides KSLW and DAL.

As for the fibroblasts, the highest migration rate was detected for KSLW and its conjugate DAL-PEG-KSLW (Fig 3), while the addition of DK5 had almost 50% inhibitory effect on the fibroblasts in comparison to the control. The rest of the peptides induced a slight stimulation of the artificial wound closure.

## Discussion

### Susceptibility of the selected microorganisms to tested AMPs

#### Antibacterial activity

The simplified definition of AMP describes peptide of amphipathic nature composed of positively charged and hydrophobic amino acids. AMPs constitute the ancient host defense molecules present in most of the living organisms and covers the wide group of compounds of high sequence diversity and complex modes of action towards pathogen's cell, influencing pathogen's metabolism, protein and DNA synthesis, disruption of membrane structure or cell wall synthesis [19].

Many empirical and computational QSAR (Quantitative Structure-Activity Relationship) researches on AMPs have been undertaken recently in order to establish which structural (amino acid composition, secondary structure of peptide, flexibility) or physicochemical properties (hydrophobicity, net-charge) are fundamental for their biological activity [20,21].

In our studies we were trying to find correlations between chemical structures of the obtained compounds and their biological properties and test the possibility of the synthesis of simple bifunctional peptides.

In general, we observed that antimicrobial properties of the native peptides (DK5, LK6, KSLW) are extremely sensitive to any structural modification. Introduction of CTEN2 peptide substantially impaired their microbicidal properties, probably due to the reduction of the net charge and hydrophobicity of the resulting conjugates. A slight improvement of bactericidal activity of DAL-containing conjugates towards Gram-positive and Gram-negative strains suggests it as better fragment of the conjugates. Surprisingly, we also found that DK5-PEG-DAL compound inhibited bacterial growth less potently than its counterpart DAL-PEG-DK5, the latter suggests that antimicrobial peptide is more likely to be effective when localized on the C—terminus of the conjugate, however this hypothesis should be verified in the future by testing other derivatives containing AMPs at two different positions. It should be also mentioned that bactericidal activity of the native peptides DK5 and LK6 was substantially weaker and strain-specific in comparison to the previously published results, where mentioned compound were active in the micromolar range towards broad range of bacteria without any substantial specificity to the Gram-nature of the tested microorganisms [22].

Unfortunately, we did not obtain any compound able to inhibit the growth of uropathogenic strain *Proteus mirabilis*. The most reasonable explanation of these results that both native monomers and designed conjugates (in spite of the presence of PEG-linker or D-Lys in case of DK5 and its derivatives) appear to be the substrates for extracellular protease ZapA (also known as mirabilysin) produced by this strain. Zap A degrades a wide group of biological substrates, including immunoglobulins, complement, cytoskeletal proteins, as well as innate antimicrobial peptides (LL-37 and hBD1) [23].

Declined susceptibility of *S*. *aureus* and *B*. *cereus* in comparison to the less pathogenic strains from the same genus, i.e. *S*. *epidermidis* and *B*. *subtilis*, respectively, could also be explained by the more developed mechanisms of virulence typical for pathogenic strains.

In case of *S*. *aureus*, it was found that this bacteria is able to produce specific enzyme staphylokinase, that chelates innate AMPs—α-defensins and thus reduce their activity [24]; proteases, that can easily degrade AMPs [25]; modifies the content of fatty acids within its membrane in growth-phase dependent manner, so that it becomes resistant to the effect of cell-penetrating antimicrobial agents [26].

As for *B*. *cereus*, besides its ability to form biofilm, that hampers effective eradication of the bacterial cells, some of its clinical isolates were found also to posses outer glycoprotein S-layer, which consists of proteinaceous paracrystalline arrays, that serves as additional protective layer and also defines its ability to adhere to the surface of epithelial cells [27].

#### Fungicidal activity

Due to the uprising incidents of infections caused by *Candida* species combined with the limited choice of approved pharmaceuticals used in treatment of the invasive mycoses, development of novel antifungal compounds is of urgent need.

Many of the species from the *Candida* genus constitute the normal human microflora, while the others become opportunistic pathogens able to cause serious skin, mucosal or even systemic infections in case of immunocompromised AIDS, cancer, transplant patients or neonates [28].

For a long time *Candida albicans* was considered as the main causative pathogen of clinically observed candidiasis, however more recent mycological studies revealed the involvement of other non-albicans Candida (NAC) in human fungemia. The most frequently identified NAC human pathogens are *Candida tropicalis*, *Candida glabrata* and *Candida parapsilosis*, and proportion of NAC species among *Candida* clinical isolates tends to increase [29]. The latter suggests the possible higher level of resistance of NAC strains to the eradication with traditional antifungal azoles, polyenes or 5-fluorocytosine [30].

In contrast to the widely studied mechanisms of antibacterial activity of AMPs, their antifungal mode of action is relatively less explored. Like in case of bacteria, fungi cell membrane is mentioned as the principle target for the peptides, especially its specific sterol and sphingolipid content [31,32,33]. However in case of some AMPs of plant or mammalian origin additional mechanisms that affects fungi viability through receptor mediated cell internalization, interaction with intracellular targets or induction of signaling cascades that finally leads to apoptosis were also observed [33].

Among the selected *Candida* strains, *C*. *tropicalis* was the most sensitive to the effect of the tested compounds, both native AMPs, as well as their conjugates inhibited fungi growth at low micromolar concentrations. In contrast to these results, MIC values for *C*. *glabrata* were substantially higher. Among the tested conjugates, the best antifungal properties were observed for DAL-PEG-DK5 or LK6, and CAR -PEG-DK5. The widest spectrum of fungicidal properties was established for KSLW, unfortunately this activity was negatively affected by conjugation with CTEN2 and DAL Previous results for KSLW published by Semlali and co-workers suggested that this peptide is also able to minimize virulence of *C*.*albicans* by decreasing its ability to adhere and grow on the surface of the epithelial cells [34]. Reduced pathogenicity of KSLW-treated *C*.*albicans* was correlated with the observed decrease in the expression of Toll-like receptors and production of human β-defensin in comparison to the wild untreated fungi. In this work KSLW was also proposed as immunomodulator of the host pro-inflammatory response, as it influenced *in vivo* production of pro-inflammatory cytokines, increasing IL-1β and decreasing IL-6 release. The latter suggests a complex way of interaction between KSLW and the cells of pathogenic fungi, thus, its conjugation with other peptides could possibly affect not only its affinity to fungi cell membrane, but also its ability to interact with other intracellular targets of pathogen.

The observed difference in the response of selected *Candida* strains to the tested compounds most probably arises from the specific morphological and physiological features of each *Candida* species (like ploidy, sexual behaviour, ability to produce hydrolytic enzymes crucial to the pathogenicity, extensiveness in biofilm formation *etc*) [28,30]. Thereby, effectiveness of treatment of candidosis, as well as the success in the design of novel antifungal compounds strongly depends on the proper identification of the causative pathogenic strain responsible for the infection.

### Analysis of pro-proliferative and pro-migratory properties

Keratinocytes and fibroblasts were utilized for *in vitro* tests due to their crucial role in the proliferation phase of wound healing. Besides proliferation, migration of fibroblasts and keratinocytes into the wounded area in the early stage of skin reparation also decides about its proper and timely healing. That is why, the additional scratch test was performed to analyze the ability of the compounds to stimulate cell migration into the area of the artificial wound.

In our study we decided to use primary dermal fibroblasts as only few transformed human fibroblast lines, reflecting properties of the primary cells, are commercially available. In case of keratinocytes we selected transformed cell line (HaCaT) as it represents a convenient *in vitro* testing model with retained physiological properties of the native keratinocytes (morphogenesis and differentiation features). Additionally, due to the lack of donor-to-donor variability, that always exists in primary cell lines derived from the skin, HaCaT cell line offers reliable and repeatable results [6,17,35–36]. As it was indicated in other studies, HaCaT keratinocytes are characterized by a similar response to peptides and hormones in comparison to primary cells[18,37–38]. The available literature also shows that HaCaT cell line is a widely accepted valuable model of normal adult human keratinocytes [6,18,37–39].

According to the obtained results, the most promising conjugates, that effectively inhibited bacterial and fungi growth (DAL-PEG-DK5 and DAL-PEG-LK6), appeared to be cytotoxic at concentrations higher than 25 μg/mL. It is worth of mentioning, that when antimicrobial peptide was introduced at the N-termini of the conjugate (like in case of DK5-PEG-DK5), it becomes non-toxic, however, at the same time its antibacterial activity substantially decays. In contrast to the negative effect of high concentration of DAL-PEG-DK5 on the cells count, it appears to be neutral or slightly stimulative at 25 μg/mL for keratinocytes and fibroblasts migration. In case of DK5 conjugated with carnosine CAR and its multiplied analogue CAR3 we did not observe any improvement of antibacterial activity, although according to the results of CD spectra analysis in 30 mM SDS, CAR-PEG-DK5 had the highest content of α-helixes that at least theoretically determines better permeabilization of bacterial membrane. Nevertheless, conjugation of CAR and CAR3 to DK5 reduced cytotoxicity of the latter towards keratinocytes at concentrations between 1 and 25 μg/mL.

Among the tested peptides, the best profile of potential wound-healing properties had the native KSLW peptide. It had both pro-proliferative effect (estimated on the basis of the increased cell metabolic activity in MTT test) on keratinocytes and fibroblasts and strong stimulatory impact on their migration to the wounded area. The best pro-migratory properties were observed for DAL-PEG-KSLW, that enhanced cell migration with the efficiency comparable to 10% FCS. Unfortunately, the antimicrobial properties of this conjugate towards pathogenic bacteria and fungi in comparison to the native KSLW was noticeably reduced.

CAR-PEG-DK5 and CAR3-PEG-DK5 conjugates induced cell proliferation when applied at their lower concentrations, like 1 or 10 μg/mL, while at 50 μg/mL became cytotoxic towards keratinocytes. The effect of CTEN2 conjugated to KSLW or LK6 was rather neutral to cells viability, but their antimicrobial properties were almost totally abolished.

It should be also mentioned, that biological activity of the native peptides CAR, CAR3, DAL and CTEN2 analyzed by means of the MTT and scratch tests towards keratinocytes and fibroblasts was not pronounced enough to speculate whether the conjugation of these peptides with the selected AMPs significantly affected their initial activity or not.

From our point of view, in order to establish the real potency of the tested peptides in the wound healing, they should be examined in the future for their ability to accelerate the closure and healing of full-thickness excisional wounds (infected or sterile) in animal model.

## Conclusion

The results presented in the article constitute the initial step of our research aimed at the design of the peptide-based bifunctional compounds with potential application in the treatment of the infected non-healing wounds. To conclude, we observed that biological activity of the native antimicrobial peptides is extremely sensitive to the structural modification, especially when they affect the net charge or hydrophobicity of the final compounds.

The best microbicidal properties were observed for compounds comprising dalargin and antimicrobial peptides DK5 or LK6, unfortunately, these conjugates exerted cytotoxicity towards keratinocytes and fibroblasts when used at higher concentrations. Nevertheless, it should be taken into account, that in comparison to the conditions of the cell culture experiments, complex biological environment can substantially reduce the potential for toxicity to human cells, maintaining the microbicidal properties unaffected. However this thesis requires further studies to generalization. Some synthetic modifications to reduce cytotoxicity of the conjugates could also be undertaken, for example, through the additional N-or C-terminal PEGylation, replacement of L-amino acids with their D-enantiomeric counterparts or unnatural analogues to minimize α-helicity and improve proteolytic stability. Though, tailoring selection towards microbial rather than eukaryotic membranes is not a simple task and should be well-considered due to the multiplicity of the factors that could affect the final result.

Among the tested conjugates we also obtained compounds, that besides antimicrobial properties, had a substantial impact on human keratinocytes and fibroblasts proliferation and migration. The best cell stimulatory effect was observed for conjugate DAL-PEG-KSLW. In spite of the fact, that its antimicrobial properties were reduced in comparison to the native KSLW peptide, it could be used for further structural optimization to improve its bacteria and fungi killing properties, for example, through end tagging with hydrophobic oligopeptide stretches or introduction of lipid moieties, that could enhance its cell-penetrating ability.

Taking into account the complexity of the wound-healing processes the most promising compounds should be examined in the appropriate *in vivo* model as they are also supposed to have antioxidative or antiinflammatory properties essential for skin regeneration, that could not be seen by means of the utilized MTT or scratch tests performed on the primary or transformed cell lines derived from human skin.

## Supporting Information

S1 File Table A: List of peptides used in the design of conjugates with characteristics of their biological properties. Figure A: CD spectra of the selected peptides described in the article.
(DOCX)
