# Supplementary material for: Synthesis and Evaluation of Biological Activity of Antimicrobial – Pro-Proliferative Peptide Conjugates
Source: PLoS One. 2015 Oct 16;10(10):e0140377. doi: 10.1371/journal.pone.0140377 (PMC4608580; doi:10.1371/journal.pone.0140377)
Supplement: S1 File — Figure A: CD spectra of the selected peptides described in the article. (DOCX) [file pone.0140377.s001.docx]

**Supporting information**

**Table A.** **List of peptides used in the design of conjugates with characteristics of their biological properties.**

| Peptide | Sequence | Biological activity |
| --- | --- | --- |
| **KSLW** | KKVVFWVKFK-NH_2_ | Analogue of peptide KSL derived from combinatorial libraries as compound with strong antibiotic activity against *Candida albicans* and wide range of bacteria species[1]. Additionally, KSLW was found to stimulate neutrophil chemotaxis and inhibit oxydative burst [2] |
| ***D*K5** | IKKILS*K*IKKLL-NH_2_ | Analogues of the naturally occurring peptide temporin-1CEb (also known as amurin-3) isolated from skin secretion of frog *Rana amurensis*. Small cationic α-helical peptides with substantial antibacterial activity towards both gram-negative and gram-positive strains and low hemolytic properties. The possible mode of action implies incorporation into bacterial plasma membrane that leads to its depolarization. [3] |
| ***L*K6** | IKKILSKIKKLLK-NH_2_ |  |
| **Dalargin (DAL)** | Y*A*GPLR | Synthetic Leu-enkephalin analogue with confirmed anti-nociceptive activity when administered intravenously encapsulated in nanoparticles [4,5] Additionally, it was found that topical application and its intraperitoneal injections promotes proliferation of fibroblasts, new capillary formation, shortens inflammatory phase in the rats model of wound healing [6]. Dalargin was also mentioned as potent drug-candidate for treatment of duodenal ulcers [7] |
| **Carnosine (CAR)** | βAla-His | Endogeneous dipeptide found in high concentrations in muscle and nervous tissues in mammals, birds, and fish [8] . Posesses cytoprotective properties due to its antioxidative (scavenging of reactive oxygen and nitrogen species), antiglicating, anticross-linking activities [9,10]. Zinc-carnosine complex was found able to stimulate synthesis of insuline-like growth factor-1 and decrease secretion of interleukin-8(IL-8) in gastric epithelium [11,12] |
| **CTEN2** | EGLEPG | The C-terminal fragment of synthetic peptide Ten2, being the part of fibronectin-III-like domain of tenascin X. Ten2 was found able to induce angiogenesis of *in vitro* cultured endothelilal cells [13]. EGLEPG sequence was chosen due to its similarity to VGVAPG hexapeptide motif identified in a large group of elastin-derived peptides, that accelerated angiogenesis in an *in vivo* model, stimulated development of new vessels, cell migration and promoted wound healing *in vitro* [14] |

**References**

1. Hong SY, Oh JE, Kwon M, Choi MJ, Lee JH, Lee BL, et al. (1998) Identification and characterization of novel antimicrobial decapeptides generated by combinatorial chemistry. Antimicrob Agents Chemother 42: 2534-2541.

2. Williams RL, Sroussi HY, Leung K, Marucha PT (2012) Antimicrobial decapeptide KSL-W enhances neutrophil chemotaxis and function. Peptides 33: 1-8.

3. Shang DJ, Li X, Sun Y, Wang C, Sun L, Wei S, et al. (2012) Design of Potent, Non-Toxic Antimicrobial Agents Based upon the Structure of the Frog Skin Peptide, Temporin-1CEb from Chinese Brown Frog, Rana chensinensis. Chemical Biology & Drug Design 79: 653-662.

4. Schroeder U, Sommerfeld P, Sabel BA (1998) Efficacy of oral dalargin-loaded nanoparticle delivery across the blood-brain barrier. Peptides 19: 777-780.

5. Das D, Lin S (2005) Double-coated poly (butylcynanoacrylate) nanoparticulate delivery systems for brain targeting of dalargin via oral administration. J Pharm Sci 94: 1343-1353.

6. Shekhter AB, Solov'eva AI, Spevak SE, Titov MI (1988) [Effects of opioid peptide dalargin on reparative processes in wound healing]. Biull Eksp Biol Med 106: 487-490.

7. Smagin VG, Vinogradov VA, Bulgakov SA, Polonskii VM, Bespalova Zh D (1984) [Clinical evaluation of hexapeptide dalargine used in the treatment of duodenal ulcer]. Ter Arkh 56: 49-52.

8. Hipkiss AR, Brownson C, Carrier MJ (2001) Carnosine, the anti-ageing, anti-oxidant dipeptide, may react with protein carbonyl groups. Mech Ageing Dev 122: 1431-1445.

9. Hipkiss AR (2010) Aging, Proteotoxicity, Mitochondria, Glycation, NAD and Carnosine: Possible Inter-Relationships and Resolution of the Oxygen Paradox. Front Aging Neurosci 2: 10.

10. Hipkiss AR (2009) Carnosine and its possible roles in nutrition and health. Adv Food Nutr Res 57: 87-154.

11. Suzuki H, Mori M, Seto K, Watanabe C, Kai A, Hasada I, et al. (2000) Zinc-carnosine chelate compound, polaprezinc, attenuates H.pylori-induced gastric mucosal leukocyte activation in Mongolian gerbils - An intravital observation of gastric microcirculation -. Gastroenterology 118: A746-A746.

12. Watanabe D, Otaka M, Mikami KI, Goto T, Miura K, Ohshima S, et al. (2004) Protective effect of zinc L-carnosine in gastric mucosa of portal hypertensive rats. Gastroenterology 126: A385-A386.

13. Demidova-Rice TN, Geevarghese A, Herman IM (2011) Bioactive peptides derived from vascular endothelial cell extracellular matrices promote microvascular morphogenesis and wound healing in vitro. Wound Repair Regen 19: 59-70.

14. Robinet A, Fahem A, Cauchard JH, Huet E, Vincent L, Lorimier S, et al. (2005) Elastin-derived peptides enhance angiogenesis by promoting endothelial cell migration and tubulogenesis through upregulation of MT1-MMP. J Cell Sci 118: 343-356.

**Figure A. CD spectra of the selected peptides described in the article**
